# Highly chromophoric Cy5-methionine for N-terminal fluorescent tagging of proteins in eukaryotic translation systems

**DOI:** 10.1038/s41598-017-12028-9

**Published:** 2017-09-14

**Authors:** Jung Min Kim, Baik Lin Seong

**Affiliations:** 1 Department of Biotechnology, College of Life Science and Biotechnology, Yonsei University, 50 Yonsei-ro, Seodaemun-gu, Seoul, 120-749 South Korea; 2 Vaccine Translational Research Center, Yonsei University, 50 Yonsei-ro, Seodaemun-gu, Seoul, 120-749 South Korea

## Abstract

Despite significant advances on fluorescent labeling of target proteins to study their structural dynamics and function, there has been need for labeling with high quantum yield ensuring high sensitivity and selectivity without sacrificing the biological function of the protein. Here as a technical advancement over non-canonical amino acid incorporation, we provided a conceptual design of the N-terminal fluorescent tagging of proteins. Cy5-labeled methionine (Cy5-Met) was chemically synthesized, and then the purified Cy5-Met was coupled with synthetic human initiator tRNA by methionine tRNA synthetase. Cy5-Met-initiator tRNA (Cy5-Met-tRNAi) was purified and transfected into HeLa cells with HIV-Tat plasmid, resulting in an efficient production of Cy5-labeled HIV-Tat protein. Based on the universal requirement in translational initiation, the approach provides co-translational incorporation of N-terminal probe to a repertoire of proteins in the eukaryote system. This methodology has potential utility in the single molecule analysis of human proteins *in vitro* and *in vivo* for addressing to their complex biological structural and functional dynamics.

## Introduction

Site-specific chemical modification of protein residues has been developed and adopted as an essential tool for studying relationship between complex biological structures, dynamics, and functions of proteins^1–3^. Complementing previous isotope labeling^4–6^, the current fluorescent labeling has been widely used in a variety of biologic research and monitoring metabolic processes^7–9^. Chemical modifications at specific sites with fluorescent molecules provide the technical platform for the analysis of conformational dynamics of proteins at single molecule level^10, 11^. Continued researches on biophysical analysis on protein have greatly diversified the tools available for protein modification with fluorescent labels. For this purpose, various approaches have been advanced for fluorescent labeling of target proteins by incorporation of natural or unnatural amino acids^9, 12, 13^. Previously reported protein-labeling involved site-directed mutagenesis of a codon into an amber or four-base codon, followed by incorporation of labeled amino acids via suppressor tRNA or initiator tRNA chemically modified with fluorescent group^14–17^. As compared with internal labeling, the N-terminal labeling is expected to affect minimally, if any, the conformation of target proteins^18, 19^; it has an advantage over the incorporation of the non-natural amino acids in the middle of open reading frame which may perturb the local conformation and significantly impair the biological activity of the modified protein. In general, fluorescent labeling of protein requires two key considerations: selection of an efficient labeling method and the biologically informative target site that can be labeled efficiently^20^. And yet, these labeling techniques in general suffer from common problems including poor site specificity, limited fluorophore location requirement and low labeling efficiency^13, 21^. The biological incorporation of initiated methionine with fluorescent groups, for example, by aminoacylation of initiator tRNA with methionine^17, 22^, followed by subsequent fluorescence conjugation, usually present low fluorescent selectivity and efficiency, often requiring cell-free translation system.

As technical advancement over prior arts, here we provided a conceptual design of the N-terminal fluorescent tagging of nascent proteins in eukaryotic system. The process involves the chemical synthesis of Cy5-labeled methionine (Cy5-Met), the aminoacylation of the human initiator tRNA (tRNAi)^23^ *in vitro* with refined chemically synthesized Cy5-Met, followed by transfection into eukaryotic cells along with plasmid encoding the target protein, resulting in N-end labeling *in vivo* by cotranslational incorporation^24^. The fluorescence of Cy5 derivatives are relatively independent of the size of alkyl substitutions, and could be detected in high quantum yields at single molecule level^25^. A mechanism linking a protein’s half-life to the nature of its N-terminal amino acid was described as the “N-end rule”^26^. The eukaryotic N-end rule distinguishes between primary, secondary, and tertiary destabilizing residues depending on the native of the N-terminal amino acid. Therefore, our the labeling process, subjected only to the violation of the N-end rule because of the chemical modification of the N-terminal methionine, could be used for specific labeling of virtually any proteins via co-translational incorporation in mammalian cells.

## Results

### Rationale for N-terminal specific labeling

**Figure 1:**
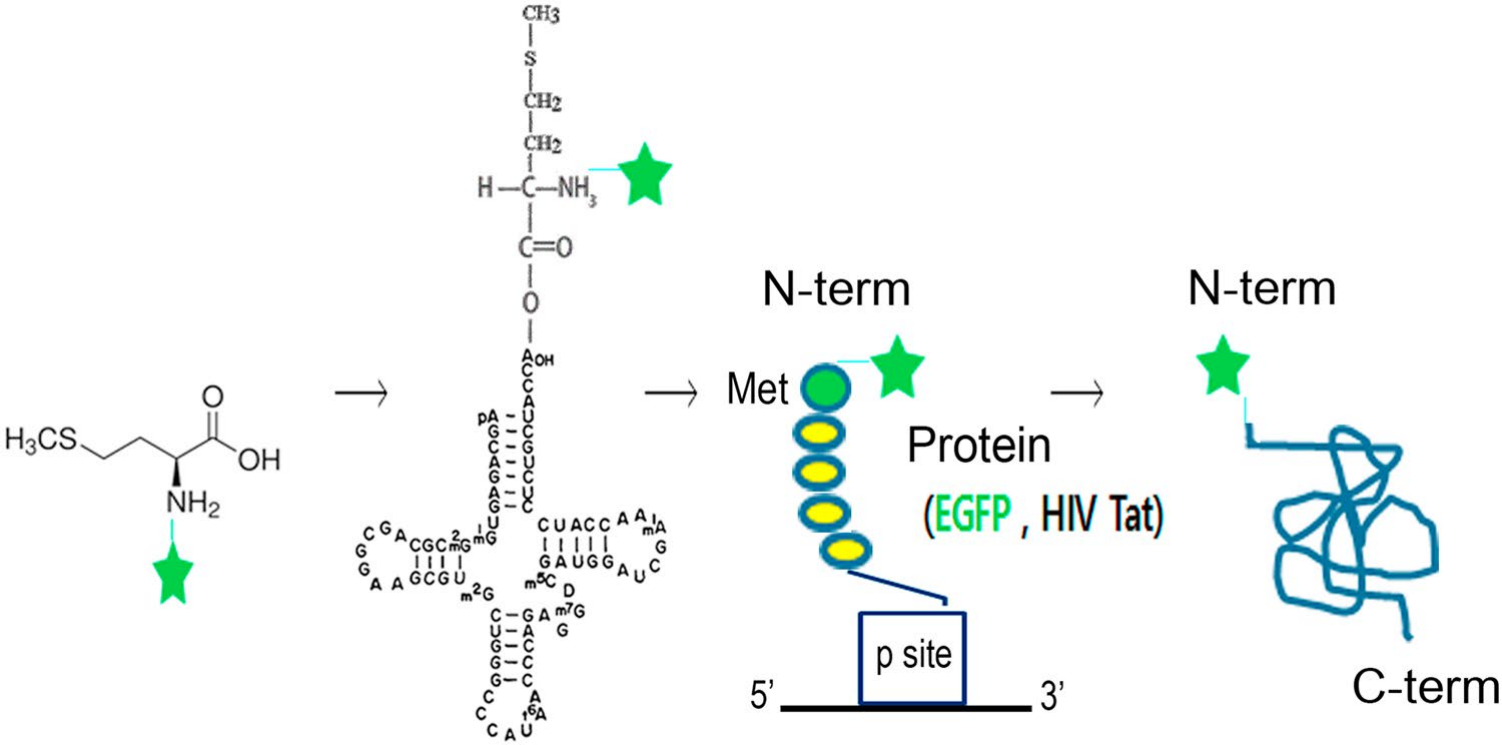
N-terminal end labeling strategy. Methionine (Met) is chemically conjugated with Cy5 () to yield Cy5-methinone (Cy5-Met). The purified Cy5-Met was used for the aminoacylation *in vitro* of human initiator tRNA (tRNAi) by MetRS to yield Cy5-Met-charged human initiator tRNA (Cy5-met-tRNAi). After purification, Cy5-met-tRNAi is then transfected into mammalian cells along with plasmids coding for target protein (EGFP or HIV Tat) for de novo N-terminal labeling.

Figure 1 shows the N-end labeling strategy through nascent protein synthesis in eukaryotic cells using human initiator tRNA (tRNAi). There are two classes of methionine tRNAs in all organisms. Of the two different methionine tRNAs (tRNA^met^) that are charged by MetRS, the initiator tRNA is specifically used for the initiation of protein synthesis^27, 28^, whereas the elongator for the incorporation of methionine into the polypeptides. Initiator tRNAs do not normally bind to the ribosomal A site, and therefore, are excluded from elongation of peptide synthesis. Thus, after methionylation, initiated tRNAs specifically bind to the initiation factor and are directed to the ribosomal P site during eukaryote protein translation system. The specific function of initiator tRNAs for the initiation is ascribed to a unique sequence and structural features that are not found in most elongator tRNAs^29^. Thus, the present system operates for site-specific labeling of N-terminal residue only, regardless of the potentially multiple methionine residues within the open reading frame. Methionine was chemically labeled with Cy5 (Cy5-Met) and then purified Cy5-Met conjugated to the initiator tRNA molecules with Met-tRNA synthetase. The fluorescence-labeled (Cy5-Met) tRNAi could be incorporated into the N-termini of proteins via nascent protein synthesis after transfection into HeLa cells (Fig. 1). Our experiments utilized two model proteins, EGFP and HIV-1 Tat protein^30, 31^. HIV-1 Tat protein was included as a model of choice because the protein does not carry any internal methionine residues in its full sequence, and therefore, any labeling of methionine could be ascribed specifically to the N-terminus (Supplementary Fig. S1).

### Analysis of purified fluorescent-labeled methionine and the labeled human initiator tRNA

**Figure 2:**
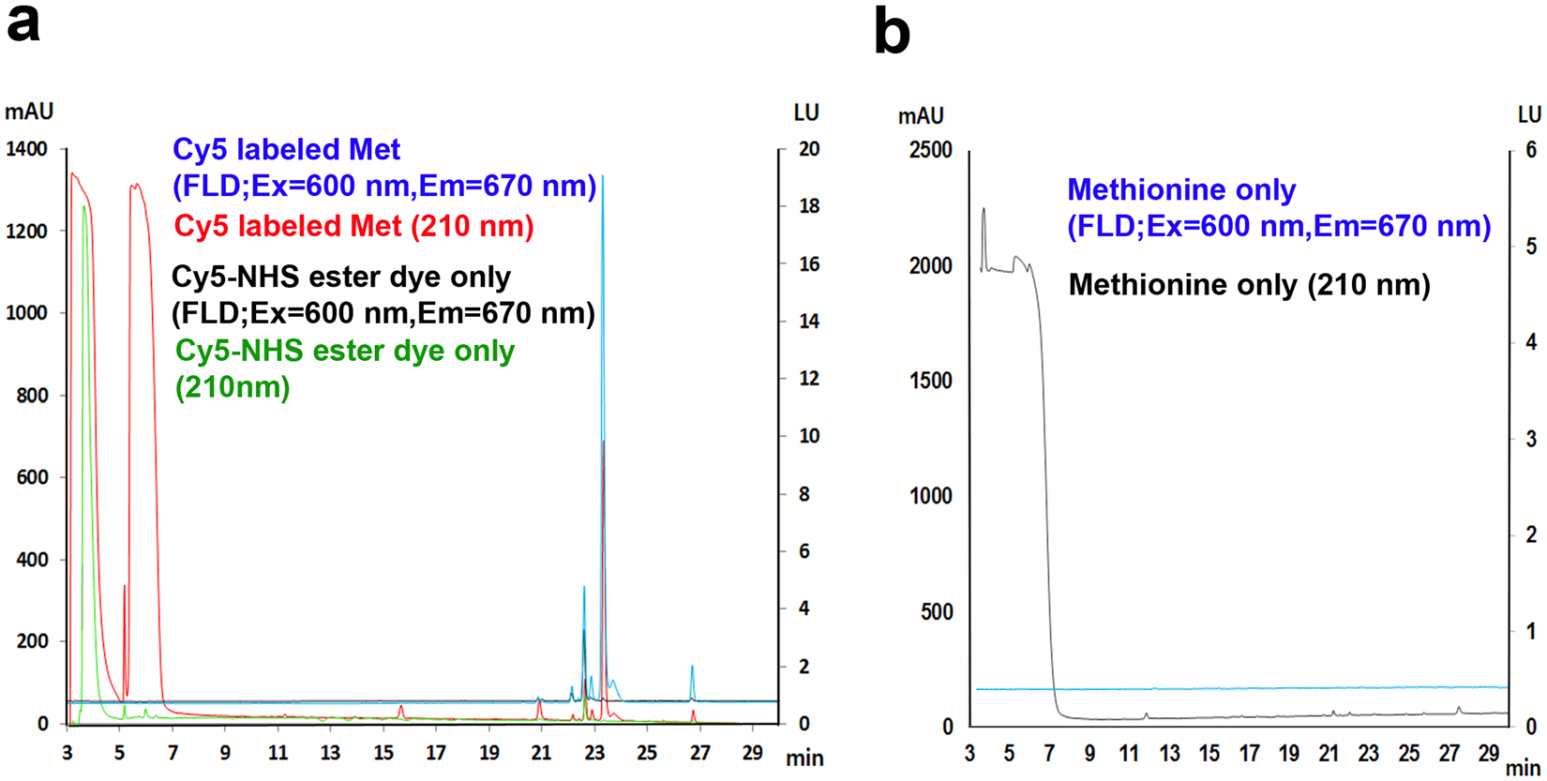
Purification of fluorescently labeled L-methionine. Fluorescently labeled methionine (Cy5-Met) was purified by high-performance liquid chromatography (HPLC) with Ultra Violet (UV) (210 nm, red line) and fluorescence detection (blue line, excitation 600 nm/emission 670 nm). (**a**) Reversed-phase HPLC and mass spectral data for Cy5 labeled methionine (Cy5-Met; blue line, FLD (excitation 600 nm/emission 670 nm); red line, UV (210 nm)), Cy5 –NHS ester dye only (Cy5) is shown in green line (210 nm) and black line (FLD (excitation = 600 nm, emission = 670 nm)). Cy5-Met peak (retention time: 23.5 min) was collected for further analysis by HPLC. (**b**) HPLC analysis of unlabeled L- methionine (Met) showing major peaks in retention time <10 min (black line; UV (210 nm) detection) without any fluorescence (blue line; FLD detector).

**Figure 3:**
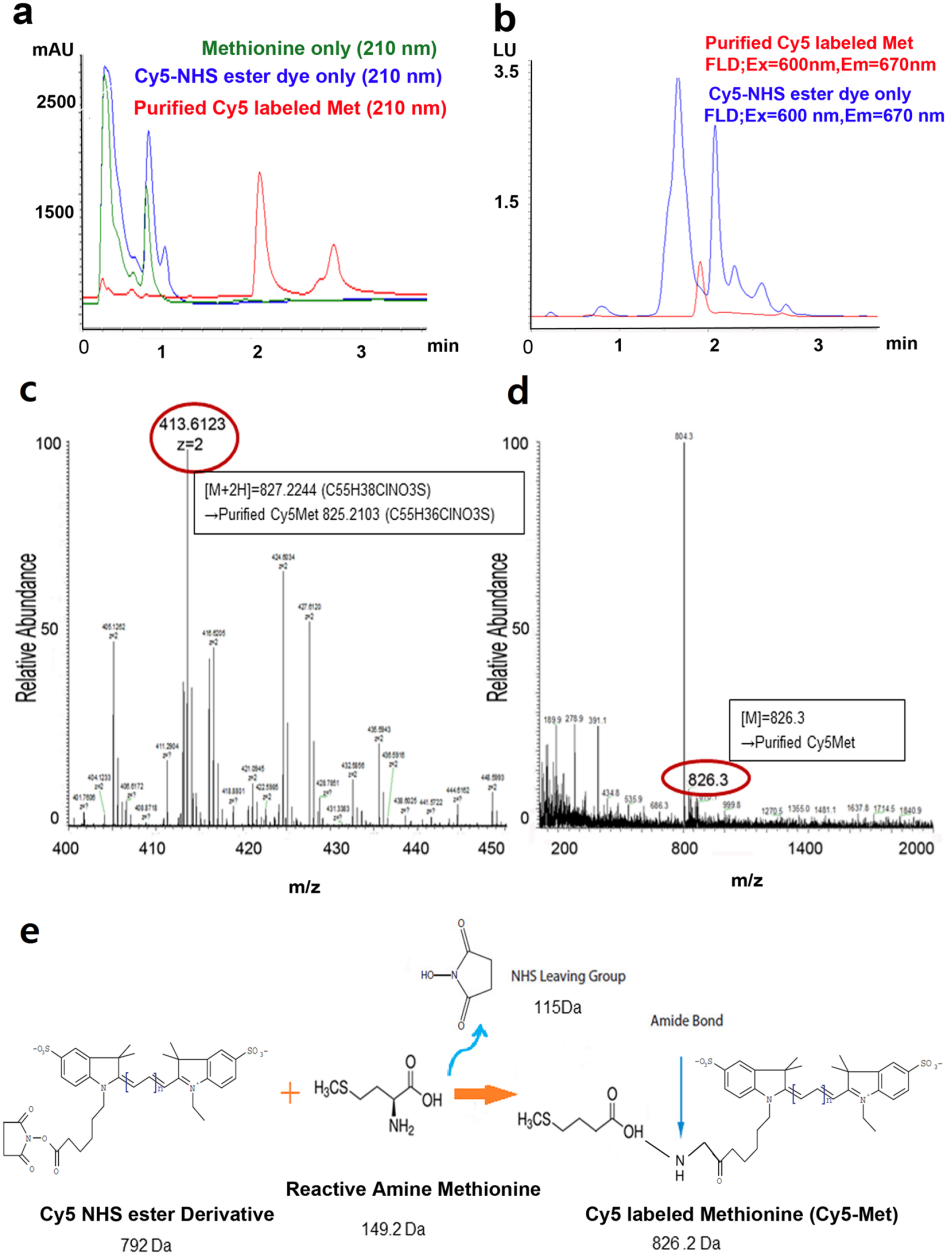
Analysis of purified fluorescently labeled L-methionine by UPLC and LC-Mass. Purified Cy5-Met in Fig. 2 was further analyzed by UPLC (**a**,**b**). (**a**) The distinct peak of Cy5-Met (826.3 Da) at 1.8 min reflects its clear separation from the reactants L-methionine (green) and Cy5 (blue), respectively. (**b**) Overlay of UPLC profiles, obtained by FLD, of purified Cy5-Met (red) and Cy5 dye alone (as a control) (blue). The purified Cy5-Met was also analyzed by LC-Mass (high resolution (**c**) and low resolution (**d**), respectively, using UV (210 nm) and fluorescence detection (FLD). (**e**) Chemical reaction for the synthesis of Cy5-Met.

The Cy5-Met was purified by reversed-phase HPLC chromatography (Fig. 2a,b) based on the UV absorbance (210 nm) and the fluorescence with the excitation and emission at 600–620 nm and 670 nm, respectively. The Cy5-Met (shown in red line (210 nm); RT (retention time) of 23.5 min) (Fig. 2a) was separated from free methionine (shown in black line (210 nm); RT < 8 min) (Fig. 2b) and Cy5 NHS Ester (Succinimidyl Ester) dye (shown in black line (FLD; Ex = 600/Em = 670); RT of <23 min) (Fig. 2a). Thus, the major peaks at specific retention time represent unlabeled methionine (Met) (210 nm; black line) (Fig. 2b), Cy5 NHS Ester dye alone (210 nm; green line) (Fig. 2a), and Cy5-Met (FLD; Ex = 600/Em = 670; blue line) (Fig. 2a). The Cy5-Met, corresponding to the highest peak at 23.5 min, was collected. The peak fraction was further fractionated using analytical reverse-phase UPLC (Fig. 3). The readout confirmed that the purified Cy5-Met is well separated from L-methionine alone and Cy5 dye (Figs 2,3 and Supplementary Fig. S7).

**Figure 4:**
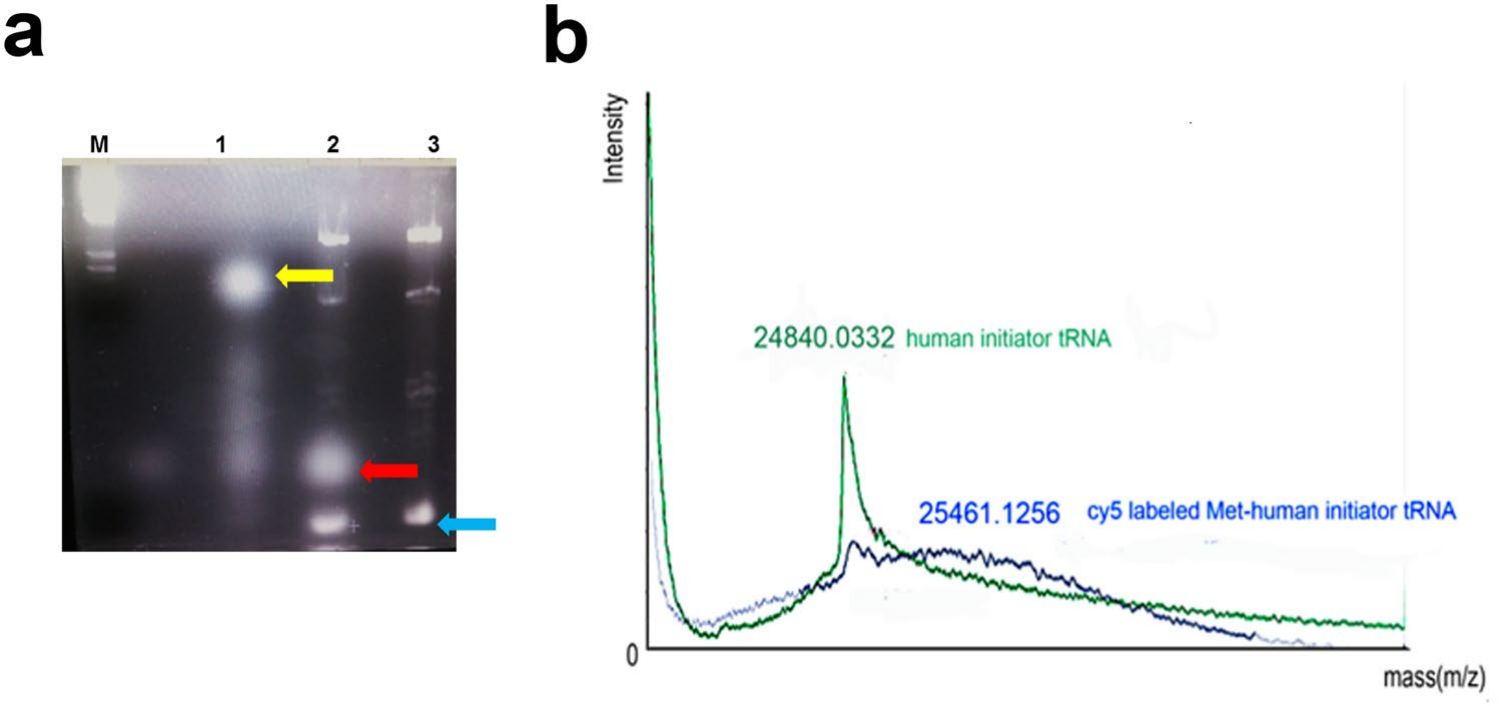
Analysis of fluorescently labeled initiator human tRNA. (**a**) Cy5–NHS ester dye only (lane 1, orange arrow) and purified Cy5-Met-charged human initiator tRNAs (Cy5-Met tRNAi) (lane 2, red arrow) visualized on a 2% agarose gel with a UV-irradiation detection. Human initiator tRNA (lane 3, blue arrow) is shown as a negative control (M: Size marker). (**b**) MALDI-TOF/TOF analysis of human initiator tRNAs (tRNAi) (green) and purified Cy5-Met tRNAi (blue).

The purity of Cy5-Met (peak fraction at RT 23.5 min) was further confirmed by subsequent UPLC analysis. The major peak with fluorescence (RT 1.9 min in Fig. 3b) represent Cy5-Met, which showed different elution profile as compared to the reactants, methionine or Cy5 ester dye (Fig. 3a,b). To further verify the quality of purified Cy5-Met, the peak fraction (RT of 23.5 min) in Fig. 2 was collected, and then subjected to liquid chromatography-mass spectrometry (LC-MS). The LC-MS data of methionine (m/z 149.21) eluted at RT 0–2 min (Fig. S7a) is different from that of purified Cy5-Met at corresponding RT (Fig. S7b), which was almost identical to the buffer only control (Fig. S7c). The Mwt of purified Cy5-Met was estimated to be 826.3 Da (low-resolution mass; RT of 11–12 min) and 827.2 Da (413.6 × 2 Da) (high-resolution mass; RT of 9–10 min) (Fig. 3c,d, respectively). The molecular mass of Cy5-Met is in good agreement with the chemical reactions involved (Fig. 3e). The concentration of Cy5-Met from the purified fraction was calculated using the linear equation of the calibration curve (data not shown). Human initiator tRNA^32^ was produced by *in vitro* transcription (see Methods) and then conjugated with the purified Cy5-Met using human MetRS followed by 2% agarose gel imaged with a Ultra Violet (UV)-irradiation device (Fig. 4a). The purified Cy5-Met-charged human initiator tRNAs (Cy5-Met tRNAi) were then analyzed by MALDI-TOF^33^, and compared with human initiator tRNA before methionylation (Fig. 4b). The differences in molecular weight reflect the purified Cy5-labeled methionine moiety added at the 3′-end of human initiator tRNA.

### Analysis of EGFP labeled with engineered human initiator tRNA

**Figure 5:**
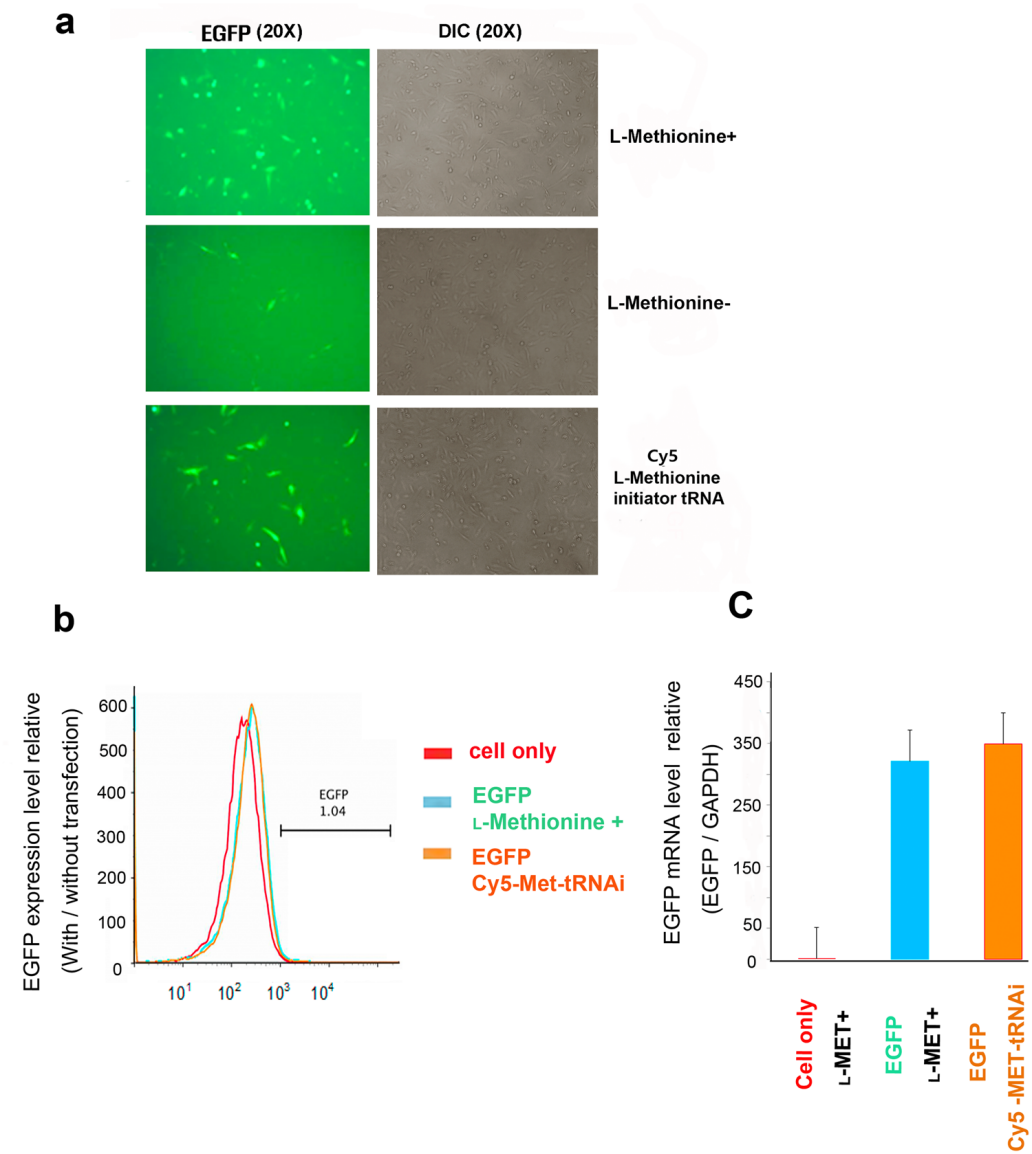
Cotranslational incorporation of modified methionine into EGFP in HeLa cells. EGFP expression was compared in cells grown under various media conditions using fluorescence microscopy. FACS analysis and qRT-PCR were used to quantitate the fluorescence and mRNA level, respectively. (**a**) Fluorescence and differential interference contrast images of EGFP expression in HeLa cells grown under the indicated conditions: medium lacking L-methionine (L-Methionine^-^), medium containing L-methionine (L-Methionine+) and methionine-free culture media supplemented with purified Cy5-Met-charged human initiator tRNAs (Cy5-Met tRNAi). Differential interference contrast images were obtained at 20× magnification. (**b**) FACS analysis was performed with EGFP cells**:** cell only (red), methionine positive media (blue), and purified Cy5-Met tRNAi in L-methionine-free media (orange). EGFP expression levels are shown as relative to cells without transfection. (**c**) EGFP mRNA levels, quantified by RT-PCR and normalized by the level of GAPDH as house-keeping gene, are shown as the mean of three independent experiments (standard deviation shown in bar).

To test whether the purified Cy5-Met-charged human initiator tRNAs (Cy5-Met tRNAi) is functional, EGFP was employed as a test protein in mammalian cells. After cotransfection of HeLa cells with Cy5-Met tRNAi and EGFP plasmids, the EGFP positive cells were monitored using fluorescence microscopy (Fig. 5a). Both the methionine-free (met−) media and the methionine-containing media (met+) were used as controls for EGFP expression. The EGFP expression in met− media was weak, if any, probably reflecting the initiation of translation by low-level endogenous met-initiator tRNAs. In contrast, the EGFP-expressing cells were greatly increased in met+ media (Fig. 5a). Notably, when the met− media was supplemented with purified Cy5-Met tRNAi, distinct EGFP-expressing cells were observed, similar to that observed in met+ media. The dependence of EGFP expression on methionine is also reflected in the EGFP specific mRNA level (data not shown). Lower level of EGFP protein and mRNA in the methionine-free (met−) media could be due to reactive oxygen species (ROS) with anabolic stress of methionine metabolism under methionine condition^34–36^. Previous studies indicated that methionine-deficient diet affects mRNA production in stress resistance^37^. We then further identified and quantified EGFP-expression cells by FACS analysis and their mRNA levels by qRT-PCR (Fig. 5b,c). An overlay of FACS data shows that the EGFP expressing cells by exogenously added Cy5-Met tRNAi was comparable to that of met+ control (Fig. 5b). Moreover, the EGFP-specific mRNA level was similar between met+ control and purified Cy5-Met tRNAi transfection as examined by qRT-PCR (Fig. 5c). The results showed that active initiation of translation could be effected by exogenous fluorescently labeled tRNAs introduced by transfection.

### Analysis of N-terminal end labeling of Tat protein

**Figure 6:**
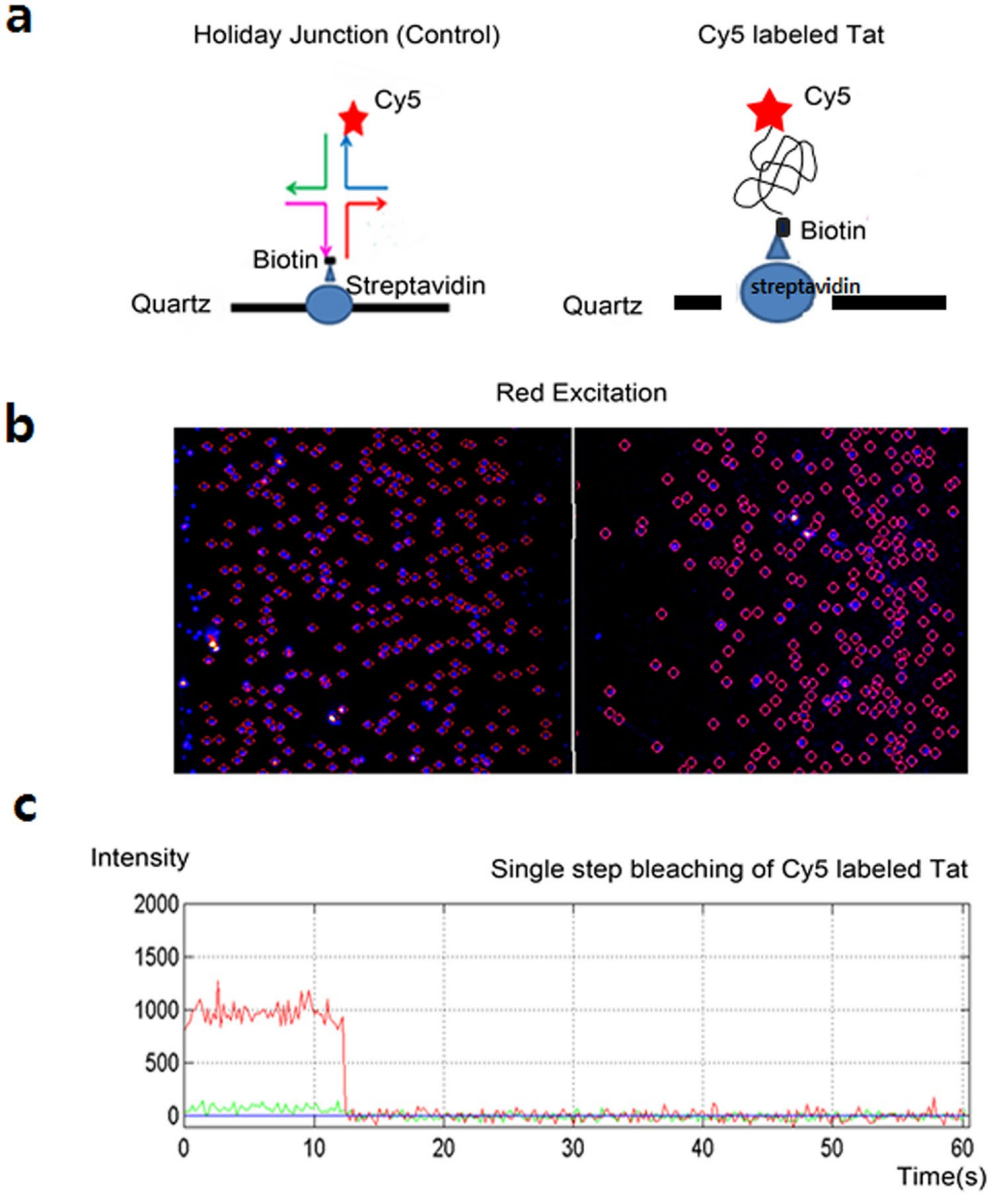
Detection of the Cy5 labeled HIV Tat protein at a single molecule level. Schematic diagram of the single-molecule Holiday Junction (positive control, left) and Cy5 labeled Tat proteins (sample, right) was immobilized quartz slides. Cy5 labeled spots (blue circle spots, right) is shown as a TIRF image of immobilized Cy5 labeled Tat proteins on the surface. Scale bar, 5 μm. The single Cy5 dye bleaching trace (red line) observed for the Cy5 labeled Tat proteins in the time-fluorescent intensity TIRF image.

The human immunodeficiency virus (HIV) Tat protein^38, 39^ does not possess any methionine within the open reading frame (ORF) (Supplementary Fig. S1), allowing us to test the specific N-terminal labeling by purified Cy5-Met-tRNAi. The HIV Tat was modified as to carry a hybrid tag composed of C-terminal AVI tag (GLNDIFEAQKIEWHE; 15 amino acids in length) and 6X His residues, for Neutravidin capture after biotinylation and Ni-affinity purification, respectively. HeLa cells were transfected with HIV-Tat plasmid and purified Cy5-Met-tRNAi and incubated in met− media for 24 h. The proteins were obtained from 4 × 10^8^ HeLa cells and the amount of labeled proteins was compared and quantified by reverse phase UPLC (Supplementary Fig. S4). Tat protein was expressed and labeled both in the presence of purified Cy5-Met and purified Cy5-Met-tRNAi. The efficiency of Cy5 labeling of Tat protein is much higher for Cy5-Met-tRNAi (~0.9 L.U.) as compared with that of Cy5-Met (~0.4 L.U.), suggesting that prior labelling as fully aminoacylated tRNA *in vitro* indeed greatly enhances the labelling efficiency of target protein. Distinctive peak at 7.8 min representing Cy5-Met-tRNAi mediated labeling was collected and were subjected to further analysis (Fig. 5). The LC-Mass analyses positively identified this peak at 7.8 min as Tat protein (Supplementary Fig. S4) which was further confirmed by Western blot (data not shown). To verify the labeling, single molecule imaging was performed using Cy5: biotin double labelled Tat on polyethylene glycol (PEG)-coated quartz slides. Figure 6 shows a representative fluorescence single-molecule image that was processed with an IDL script to identify Cy5 spot and to extract Cy5 fluorescence intensity of individual spots (shown in red circles). The single-molecule fluorescence images were successively taken using total internal reflection fluorescence (TIRF) microscope^8, 40^. A fluorescent bead image was used to create a map and fit to a calibration file, which was then used for mapping to a corresponding sample spot position. The Cy5 labeled Tat proteins were well imaged, favorably comparing with the Holliday junction as a control (Fig. 6b). The single dye bleaching event was also observed for the Cy5-Tat protein in TIRF image (Fig. 6c), confirming that each fluorescent spot corresponded to labeled Tat protein.

Cy5 labeled HIV Tat protein for N-terminal fluorescent probe activity in HeLa cells was evaluated by analytical fluorescence spectrum (Supplementary Fig. S5). Detection of the N-terminally fluorescence-labeled Tat protein was performed using Neutravidin coated 96 well plates. Tat protein containing a C-terminal AVI-tag was recovered from HeLa extracts using Ni-affinity spin columns. The fluorescence of Cy5: biotin double labelled Tat was detected using Red laser (620–670 nm), with relevant control of ATTO 565-Biotin (Sigma) (green laser; 565–590 nm) (Supplementary Fig. S6). The data show that highly chromophoric double labeling the N-terminal end by fluorescently modified initiating amino acid and the C-terminal end by biotin, respectively, could be efficiently utilized as probes for structural analyses of target proteins at single molecule level.

## Discussion

Studies on the structural dynamics and function of proteins at single molecule level are greatly benefited by specific labeling of target proteins at high sensitivity, selectivity without sacrificing their biological function. In general, fluorescent labeling of amino acids involves two key considerations: selection of an efficient labeling method and protein that can be labeled at biologically informative sites^11, 41^. Labeling at the extreme end is favored over internal labeling, which usually requires incorporation of non-canonical or non-synonymous amino acids that may perturb the local conformation significantly impairing the biological activity of the modified protein^15, 19, 21, 42^. Here, we provided an efficient N-terminal fluorescent tagging of proteins with highly purified chromophoric methionine derivatives. This approach could be utilized for N-terminal labeling of any proteins of interest considering the universal requirement of methionine and specific initiator tRNA in translational initiation. Most of N-terminal labeling methods have been advanced in bacterial system^17, 42^, based either on natural formyl-methionine initiator tRNA^14, 22^ or engineered initiator suppressor tRNAs^15^. N-terminal labeling was also reported in mammalian system, based on mutant MetRS of *E*. *coli* origin that has site selectivity on initiator, but not on elongator tRNA^19^.

Here, we provide a highly refined system by which N-terminal labeling could be achieved in mammalian system. Basically, the present process involves three steps; (i) chemical synthesis of Cy5-Met in high purity, (ii) coupling *in vitro* of Cy5-Met to the 3′-end of synthetic human initiator tRNA by MetRS, (iii) transfection of purified Cy5-Met-charged human initiator tRNAs into mammalian cells along with plasmid expressing target protein (Fig. 1). The labelled protein could be further purified for subsequent studies on single protein dynamics *in vitro* (Supplementary Fig. S2 and Fig. 6). The Cy5-Met was purified by HPLC (Fig. 2), its purity confirmed by LC-Mass analysis (Fig. 3), and subsequently the aminoacylation of initiator tRNA was confirmed by MALDI-TOF/TOF analysis (Fig. 4). The quantum yield, with respect to specific labeling of methionine, was not precisely measured here, but is expected to be comparable to previous report^26^. After cotransfection, cotranslational incorporation of the chemically modified methionine residue into HIV-Tat was analysed by combined SEC-HPLC and UPLC analyses (Supplementary Fig. S2). The successful incorporation was further confirmed with the EGFP reporter system (Fig. 5). It should be noted, however, that in case of EGFP reporter, although the fluorescence and mRNA transcription could be observed (Fig. 5), the translational initiation could be either from endogenous met-initiator tRNA with low yield (Supplementary Fig. S3) or from purified Cy5-Met-tRNAi as desired. However, unequivocal evidence for direct cotranslational incorporation could be deduced from HIV-Tat (Fig. 6)^43^. The HIV-Tat was of choice as target protein since the protein does not contain any methionine in the whole coding region, and therefore, any labeling could be ascribed to N-terminal specific (Supplementary Fig. S1).

For the initiation codon mediated N-terminal labeling of target proteins, considerations should be given to potential N-terminal processing of nascent polypeptides. Unlike in eubacteria, the formylation of initiator methionyl-tRNA by methionyl-tRNA formyltransferase is not required for the initiation of protein synthesis in eukaryotic systems^44^. Although methionine aminopeptidase catalyzes the hydrolytic cleavage of N-terminal methionine from newly synthesized polypeptides, the chemically modified methionine as designed in this report may resist the cleavage^45, 46^, resulting in stable labeling suitable for biochemical studies. Considering the N-end rule that defines the relationship between protein stability and amino-terminal residue, the labeled protein is expected to have relatively long half-life; first, N-terminal Met belongs to the class of stabilizing residues, and second, the chemical modification may interfere with the interaction with the ubiquitin dependent proteosomal machinery into subsequent degradation^26, 47, 48^. Thus, our method is expected to complement pre-existing labeling methodologies as essential tools for introducing fluorescence probes into defined positions in target protein as powerful approaches for studying their conformational dynamics by the single-molecule analysis technique^24, 49, 50^. Furthermore, the purified Cy5-Met, when supplemented in culture media, is expected to provide a metabolic N-end labeling method using pre-existing endogenous initiator tRNA and MetRS in mammalian cells, whereas it is excluded from internal labeling due to the masking of the amino group. Therefore, further extending prior arts on toolbox for protein design, our method contribute to studying the conformational dynamics of proteins at single molecule level, for understanding their physiological role and pathological consequences in related human diseases.

## Methods

### Construction of expression plasmid

**E**nhanced green fluorescent protein (EGFP) and HIV-1 transcription activator protein Tat containing Avitag and 6X His tag were used as model proteins for evaluation of the present labeling method. EGFP expression plasmid was constructed by conventional PCR sub-cloning of EGFP gene of pEGFP-N1 (Clonetech, Mountain View, CA, U.S.A.) into pcDNA3.1(+) (Invitrogen, Carlsbad, CA, U.S.A.). HIV Tat plasmid was constructed as follows: the HIV-1 Tat gene was selected from the HIV-1 complete genome (GenBank ID: NC_001802), chemically synthesized (GenScript), and used as a template for PCR amplification. Forward primer was 5-GGATCCATGGAGCCAGTAGATCCTAGACTAGAG-3, and backward primer was 5-GAA TTC TTA TTC GTG CCA TTC GAT TTT CTG AGC CTC GAA GAT GTC GTT CAG ACCCGCGG-3. *BamH*I and *EcoR*I restriction sites were generated using standard restriction cloning methods and the fragment was inserted into pcDNA3.1+ (Invitrogen).

### Preparation of fluorophore-conjugated methionine with high purity

Fluorophore-conjugated methionine was prepared by conjugation of the alpha amino group of L-methionine with Cyanine5 (Cy5) NHS ester dye (GE Healthcare, Little Chalfont, UK). Briefly, 1 mg of Cy5 NHS ester dye was dissolved in 0.1 ml of 62.5 mM sodium tetraborate buffer (pH 8.5). After adding L-methionine in saturation, the mixture was incubated overnight at 4 °C. Fluorophore (Cy5)-conjugated methionine (Cy5-Met) was purified by reverse-phase chromatography on an Agilent HPLC system using a C18 column (4.6 × 250 mm). The solvent system consisted of solvent A (0.1% trifluoroacetic acid [99.5% purity; Sigma]) and solvent B (0.1% TFA in acetonitrile) with a linear gradient for solvent B of 20–100% over 40 min at a 1 mL/min flow rate. Purity (>95%) was confirmed by analytical reverse-phase HPLC. The fractions containing Cy5-conjugated methionine (purified Cy5-Met) were dried by freeze-drying. The resulting powder was reconstituted by adding minimum volume of water (approximately 50 μl). And then, the purified Cy5-conjugated methionine (purified Cy5-Met) was analyzed by reverse-phase chromatography on a 1290 Infinity Ultra-High-Performance liquid chromatography system (Agilent Technologies, Santa Clara, CA, U.S.A) equipped with an EclipsePlus C18 column (RRHD, 1.8 μm; 2.1 × 50 mm) and UV and FLD fluorescent detection systems. The solvent system consisted of solvent A [0.1% trifluoroacetic acid (TFA) (99.5% purity) in water] and solvent B [0.1% TFA in acetonitrile] with a linear gradient of 20–80% solvent B over 40 min at a flow rate of 0.5 mL/min.

This was used in the subsequent aminoacylation of human initiator tRNA. The content of unconjugated methionine in the preparation of Cy5-conjugated methionine was assessed by liquid chromatography-mass spectrometry (LC-MS) (Ultimate 3000 RS UHPLC, Thermo Scientific LTQ Orbitrap XL, Thermo Fisher Scientific, Waltham, MA, U.S.A.) equipped with an Acquity UPLC C18 column (BEH C18 2.1 × 100 mm, 1.8 μm, Waters, Milford, MA, U.S.A.). The solvent system consisted of solvent A [0.1% TFA (99.5% purity) in water] and solvent B [0.1% TFA in acetonitrile] with a linear gradient of 0–100% solvent B over 22 min at a flow rate of 0.40 mL/min. Mass spectrometric analyses were performed using a ThermoFinnigan LCQ Deca XP plus ion trap mass spectrometer, with ESI interface (Thermo Fisher Scientific, Waltham, MA, U.S.A.).

### Production of fluorophore-conjugated methionyl human initiator tRNA

Human initiator tRNA was prepared *via* T7 RNA polymerase-mediated *in vitro* transcription of the PCR product containing the gene of human initiator tRNA^27, 32^. The PCR product containing the gene of human initiator tRNA was prepared according to the method previously described^51, 52^. T7 RNA polymerase was obtained from Promega (Madison, WI, U.S.A.). *In vitro* transcription of linearized plasmid followed by 2% agarose gel imaged with a UV-irradiation device (data not shown). For preparation of fluorophore-conjugated methionyl human initiator tRNA, the *in vitro* transcribed human initiator tRNAs were aminoacylated with the purified Cy5-Met using purified human methionyl-tRNA synthetase (MetRS)^52^ in the reaction buffer containing 30 mM Hepes (pH 7.4), 100 mM potassium acetate, 10 mM magnesium acetate, and 100 mM ATP. After incubation at 37 °C for 10 min, 0.1 volumes of 2.5 M NaOAc (pH 4.5) were added to the reaction mixture. The tRNA was then extracted with phenol saturated with 10 mM NaOAc (pH 4.5) and precipitated with ethanol. After washing with 70% ethanol, the pellet was dissolved in RNase-free water. The synthesis of Cy5-conjugated methionyl human initiator tRNA was analyzed by both agarose gel electrophoresis analysis and mass spectrometry using a MALDI-TOF/TOF 5800 system (AB SCIEX, Concord, ON, U.S.A.) operated in linear positive ion mode at the Korea Basic Science Institute (Seoul)^33^. The matrix solution consisted of 3-hydroxypicolinic acid (50 g/L in H_2_O) and diammonium citrate (50 g/L in H_2_O). The mass accuracy was estimated within about ±0.2%, using bovine serum albumin in sinapinic acid matrix as external calibration.

### Co-translational fluorescent labeling of target proteins

For co-translational fluorescent labeling of target proteins, co-transfection of expression plasmid of target protein and purified fluorophore-conjugated methionyl human initiator tRNA (Cy5-Met tRNAi) was performed. In brief, HeLa cells were grown in MEM without phenol red (Welgene Inc., Daegu, KOREA) supplemented with 10% (v/v) fetal bovine serum and 1% penicillin/streptomycin in a 5% CO_2_ atmosphere at 37 °C. When cells were grown at 60–90% confluence, cells were washed once with growth medium (MEM without phenol red), twice with 1× phosphate buffered saline (PBS) at pH 7.4, and once with 1× PBS (pH 7.4). The washed cells were incubated in methionine-free (met-) MEM without phenol red for about 3–6 hrs, and then transfected with 500 ng each target protein expression plasmid and 100 pmol of purified Cy5-Met tRNAi per 2 × 10^6^ cells using 2 µL Lipofectamine 2000 (Invitrogen, Carlsbad, CA, U.S.A.), and incubated 37 °C for 24 h.

### Analysis of expression levels of EGFP and Tat

Analysis of the over-expression of EGFP was performed using laser-induced fluorescence with an inverted fluorescence microscope (Olympus IX71) and by fluorescence- activated cell sorting (FACS) (BD FACSVerse flow cytometer, BD Biosciences, San Jones, CA, U.S.A.). Fluorescence images were normalized to the same intensity range. The 488 nm and 545 nm laser line were used for green and red fluorescence, respectively. The laser was focused into the channel using a 20× objective; the fluorescent signal was collected by the same lens and filtered optically. To detect green fluorescence, the green filter set U-MWIBA3 EGFP shifts free (BP460–495 BA510–550) was used. Analysis of the flow cytometric data was performed using the BD FACSuite and Flow Jo software. For quantitation by real-time quantitative polymerase chain reaction (qRT-PCR), total RNA was extracted from HeLa cells using the RNeasy Mini Kit (Qiagen, Valencia, CA, U.S.A.) according to the manufacturer’s instructions. Total RNA (0.15 μg) was converted to single-stranded cDNA using Superscript III Reverse transcriptase (Invitrogen, Carlsbad, CA, U.S.A.) and the oligo dT. The qRT-PCR was performed with 15 ng cDNA using the SYBR Green (LightCycler^®^480 SYBR Green I Master) (Roche, Indianapolis, IN, USA) and the LightCycler PCR equipment (Roche, Indianapolis, IN, USA). PCR conditions were as follows: 95 °C for 5 min, followed by 40 cycles of 10 s at 95 °C, 15 s at 56 °C, and 20 s at 72 °C. For EGFP, the oligonucleotide forward primer was: 5-GGCACAAGCTGGAGTACAAC-3 and the reverse primer was 5-ATGCCGTTCTTCTGCTTGTC-3. All measurements were normalized to the human housekeeping gene glyceraldehyde-3-phosphate dehydrogenase (GAPDH).

### Purification of His tagged Tat protein in HeLa extracts

For N-terminal Cy5-labeled protein expression, HeLa cells were plated on 6-cm dishes (2 × 10^4^/dish, Nunc, Denmark) and were lysed with FT LYSIS Buffer containing 600 mM KCl, 20 mM Tris-Cl (pH 7.8) and 20% glycerol, respectively, according to the Tansey Lab’s ultimate freeze-thaw lysis for mammalian cell protocol^53^. The lysate was centrifuged at 15,000 RPM at 4 °C for 15 min, and clarified supernatant was loaded on HisPur™ Ni-NTA Spin Columns (Pierce Biotechnology, Rockford, IL, U.S.A.)^54^. The composition of binding and elution buffers is 50 mM sodium phosphate, 300 mM sodium chloride (PBS) without 10 mM imidazole at pH 7.2 and pH 5.8, respectively. The purified protein was then further analyzed using UPLC and size exclusion chromatography on an HPLC system (Agilent Technologies, Inc., Santa Clara, CA, U.S.A.) using a Yarra SEC-2000column (300 × 7.8 mm) (Phenomenex, USA). The solvent system consisted of solvent A [150 mM sodium chloride and 50 mM sodium in 50 mM sodium phosphate (pH 6.5)], equilibrated with a linear mode for solvent B[0.1% trifluoroacetic acid (TFA) (99.5% purity; Sigma) in water]over 70 min at a 1 mL/min flow rate. The purity (>95%) of the N-terminally labeled protein was confirmed by analytical size exclusion chromatography SEC-HPLC (Agilent). Western blotting was performed with a Simple Western™ system (WES. Protein Simple, San Jose, CA) using an anti-His penta antibody (1:1000 in TBST) (Qiagen, Hilden, Germany), for detection (data not shown).

### Mass spectrometric analysis of labeled protein

The N-terminal Cy5-labeled Tat was separated by SDS-PAGE, identified by staining with Coomassie Blue, and in-gel digested. The LC-MS analysis was performed with an LTQ OrbitrapVelos mass spectrometer (ThermoFinnigan, USA). Data were acquired in data-dependent mode to simultaneous recording of full-scan mass and collision-induced dissociation (CID) spectra with multistage activation. For Tat protein peptide mapping, the CID spectra was compared to the sequence of HIV-1 Tat using Sequest (Bioworks; Thermo Electron, USA).

### Single protein fluorescence detection

Single-molecule fluorescence images were obtained in a prism type wide-field total-internal-reflection fluorescence microscope using an electron multiplying charge-coupled device (EM-CCD) camera (iXon DV887ECS-BV, Andor Technology, CT, U.S.A.), a red laser (Cy5, Exceisior-635-5c, Spectra Physics, Japan) and a program written in Visual C++ program (Microsoft, Seattle, WA). Molecular Probes® Carboxylate-modified FluoSpheres® beads (Diameter 0.2 µm, Invitrogen) permitted the calibration of a single-molecule measurement. Biotinylated polyethylene glycol (biotin-PEG-SC; MW5000, Laysan Bio, AL, U.S.A.) coated quartz slides were prepared as described previously^55^. Holliday junction (HJ) DNA strands were purchased from IDTDNA (Coralville, IA)^40^. The DNA were attached to the surface by successive additions of 40 ml of 1 mg/ml biotinylated PEG (biotin-PEG-SC; MW5000, Laysan Bio, AL, U.S.A.), 40 ml of 0.2 mg/ml streptavidin (Molecular Probes, OR, U.S.A.), and finally 40 ml of 10–50 pM biotinylated Holliday junction in 10 mM Tris:HCl (pH 7.5), 50 mM NaCl and 50 mM MgCl_2_ (T50 buffer). After washing with T50 buffer, a check was performed to confirm that streptavidin and free fluorescent background molecules were well separated from each other. The Holliday junctions and Cy5 labeled Tat protein experiments were performed at 37 °C in 20 mM Tris-HCl imaging buffer (pH 8.0) with 10 mM Tris:HCl (pH 7.5), 50 mM NaCl, and an oxygen scavenger system (1 mM Trolox, 1 mg/mL glucose oxidase, 0.04 mg/mL catalase, 0.4% [w/v] glucose (Sigma-Aldrich, St.Louis MO, U.S.A.) to slow photo-bleaching. To obtain single-molecule time traces, or dwell-times, either 300-ms or 1000-ms of EM-CCD exposure time was used. Single-molecule data analysis was performed using Matlab and IDL. The Multi-mode Microplate reader FlexStation 3 system (Molecular Devices, CA, U.S.A.) was used for the detection of the Cy5-labeled Tat proteins. The protein was serially diluted over Pierce™ NeutrAvidin™ Coated High Capacity Plates, black (Thermo Scientific, Rockford, U.S.A.) by spectrum mode (the range in 350–750 nm). For biotinylation, the purified His tagged Tat protein from HeLa extracts was incubated in a buffer containing 50 mM bicine, pH 8.3, 10 mM ATP, 10 mM Mg (OAc)_2_, the d-biotin with 0.5 units of BirA ligase (AviTag^TM^, Avidity LLC, CO, U.S.A.) overnight at 4 °C.

## Electronic supplementary material

Supporting Information
