# Supplementary material for: Highly chromophoric Cy5-methionine for N-terminal fluorescent tagging of proteins in eukaryotic translation systems
Source: Sci Rep. 2017 Sep 14;7:11642. doi: 10.1038/s41598-017-12028-9 (PMC5599622; doi:10.1038/s41598-017-12028-9)
Supplement: Supplementary file 1 — Supporting Information [file 41598_2017_12028_MOESM1_ESM.pdf]

Supporting Information

Highly chromophoric Cy5-methionine for N-terminal fluorescent tagging of proteins in eukaryotic translation systems

Jung Min Kim[1] & Baik Lin Seong[1, 2*]

[1]Department of Biotechnology , College of Life Science and Biotechnology, Yonsei University , 50 Yonsei-ro, Seodaemun-gu, Seoul 120-749, South Korea

[2]Vaccine Translational Research Center, Yonsei University, 50 Yonsei-ro, Seodaemun-gu, Seoul 120-749, South Korea

Titles of Supplementary Information

Supplementary Figure1: The construction of N-terminal tagged HIV-1 Tat protein.

Supplementary Figure2: Quantitative UPLC analysis of labeled Tat protein from HeLa cells.

Supplementary Figure3: Analysis of N-end labelled HIV Tat protein by combined SEC- H PLC and quantitative UPLC analysis of labeled Tat protein f rom HeLa cells.

Supplementary Figure4: Mass spectrum profile of predigested Cy5 labeled Tat protein.

Supplementary Figure5: Evaluation of the Cy5 labeled HIV Tat protein for N-termin al fluorescent probe activity in HeLa cells by analytical flu orescence spectrum.

Supplementary Figure6: Evaluation of the Cy5 labeled HIV Tat protein while N-terminal fluorescent probe activity as control in HeLa cells by analytical fluorescence spectrum.

Supplementary Figure7: Comparison of LC-Mass spectrum profile of the purified Cy5 labeled methionine.

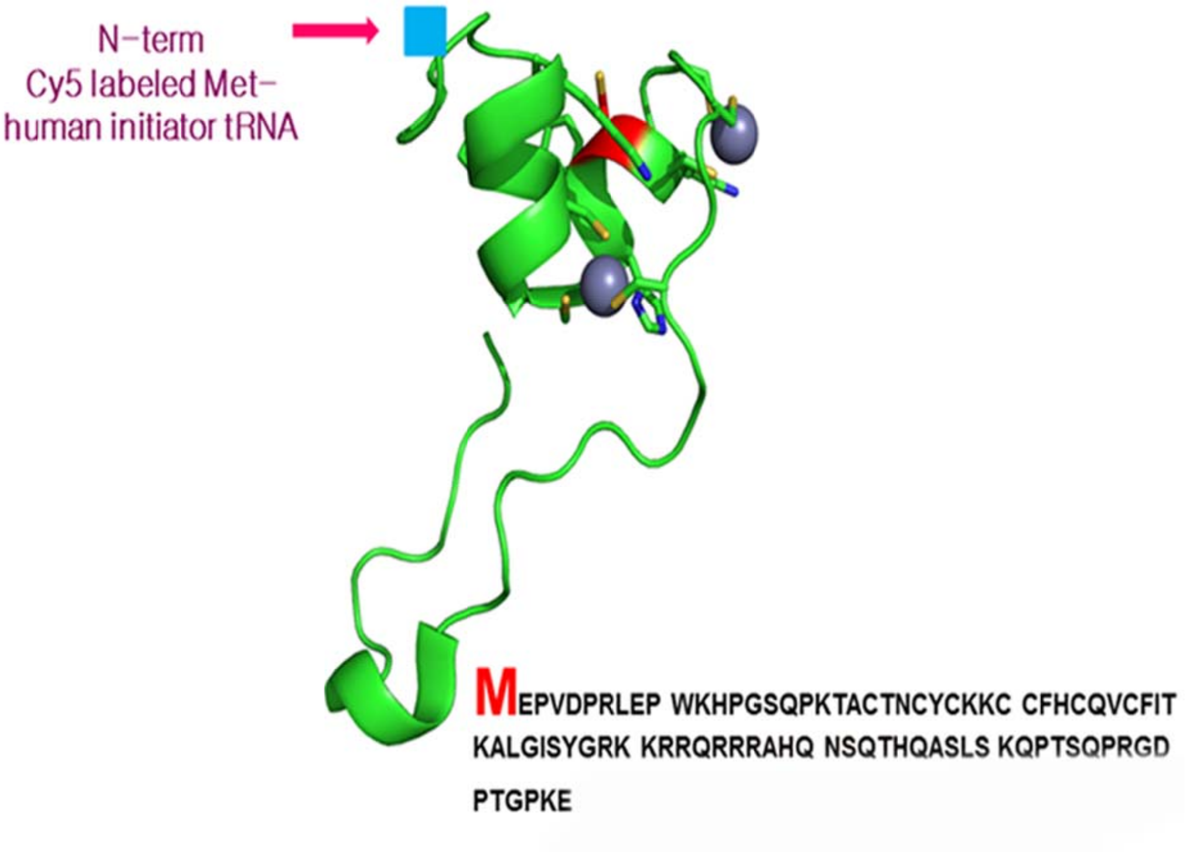

Figure S1. The construction of N-terminal tagged HIV-1 Tat protein.

Schematic description of the HIV Tat protein labeled using Cy5-Met (Cy5 labeled methionine), purified Cy5-Met and Cy5-Met tRNAi (Cy5-Met coupled with synthetic human initiator tRNA) at the N-terminus. The Cy5-labeled HIV-Tat protein was produced by Cy5-Met-tRNAi which was purified and transfected into HeLa cells.

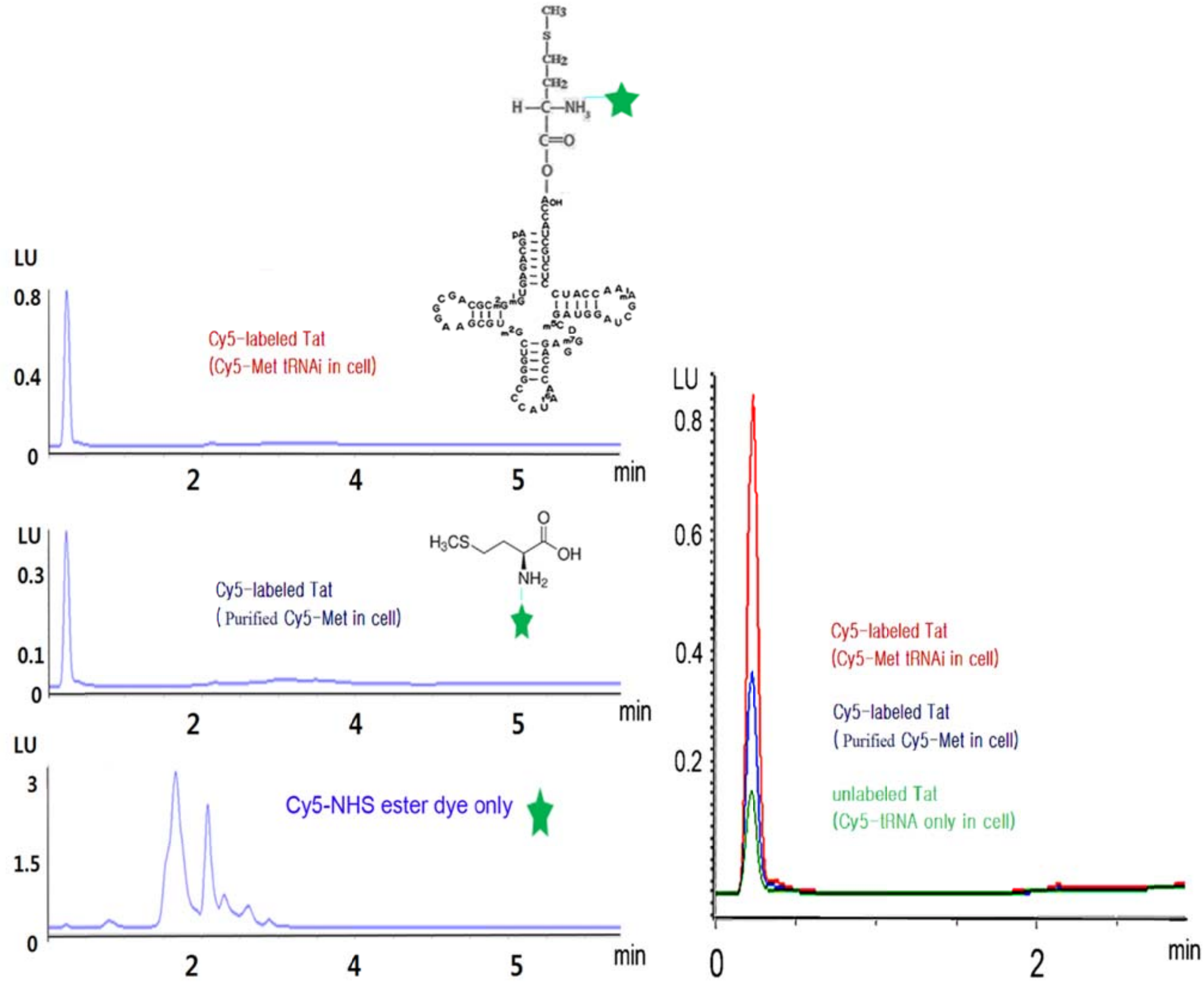

Figure S2. Quantitative UPLC analysis of labeled Tat protein from HeLa cells.

The analytical reverse-phase ultra-performance liquid chromatography (UPLC) profiles of the N-terminal fluorescence-labeled Tat protein. Compared to the peaks from amine-reactive Cy5 alone at 2 min, Cy5-labeled Tat protein from HeLa cells is shown with the highest peak at 0.8 L.U. using a fluorescence detector (Cy5-Met tRNAi; purified Cy5 labeled methionine conjugated human initiator tRNAs, Purified Cy5-Met;Cy5 labeled methionine). The proteins were isolated from 4×10^8 HeLa cells (2×10^4/ 6 cm, Nunc; Denmark) and subjected to analysis.

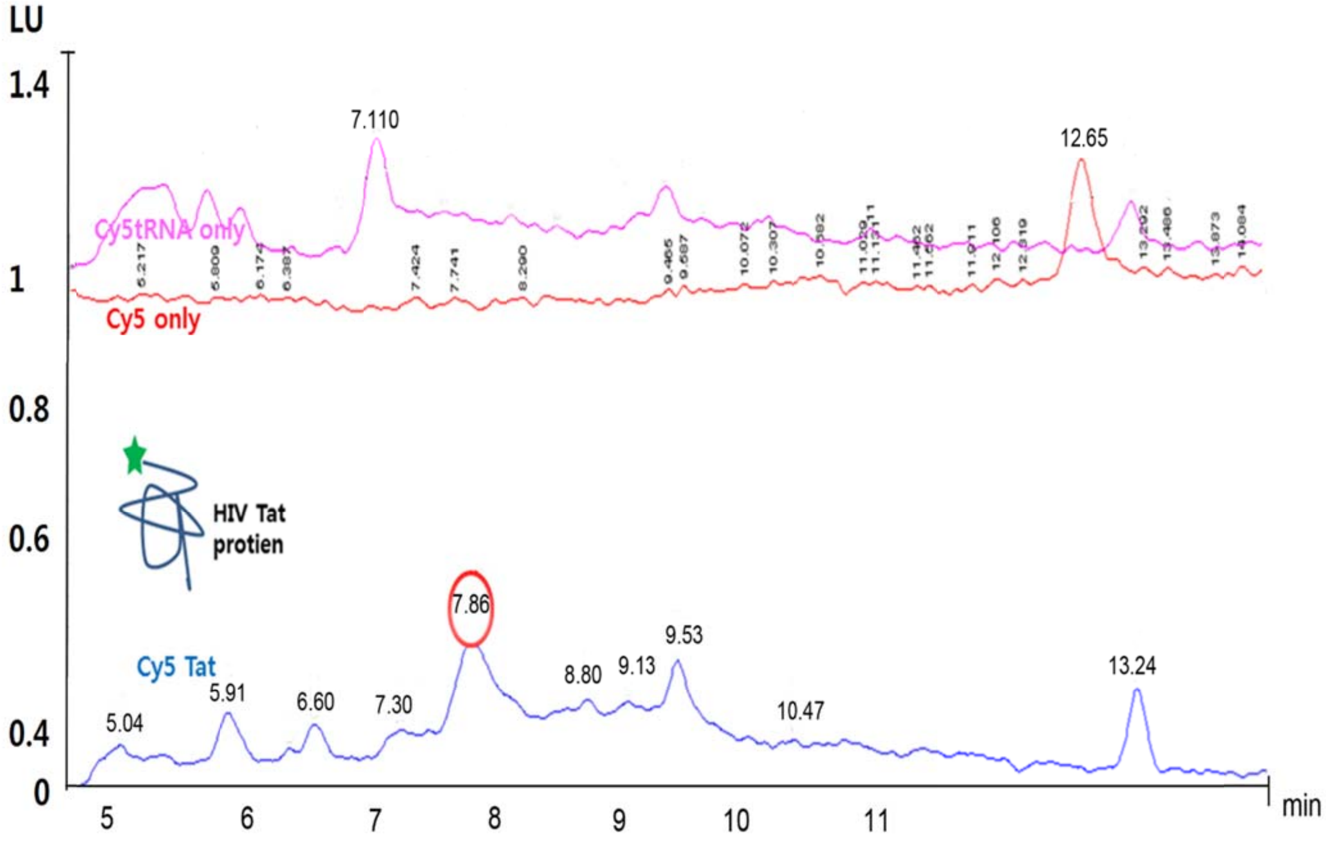

Figure S3. Analysis of N-end labelled HIV Tat protein by combined SEC- HPLC and quantit ative UPLC analysis of labeled Tat protein from HeLa cells.

The analytical UPLC profiles of the N-terminal fluorescence-labeled Tat protein. Size exclusion chromatography of N-terminally fluorescence-labeled Tat shows highest peak at 7.865 min. The peak fraction were pooled and subjected to LC-MS analysis (Fig.S 4). The SEC- HPLC; Cy5-Met-tRNAi mediated labeling (FLD-blue line), Cy5-Met-tRNA complex only (FLD-pink line), and Cy5-NHS ester dye only (FLD-red line), respectively.

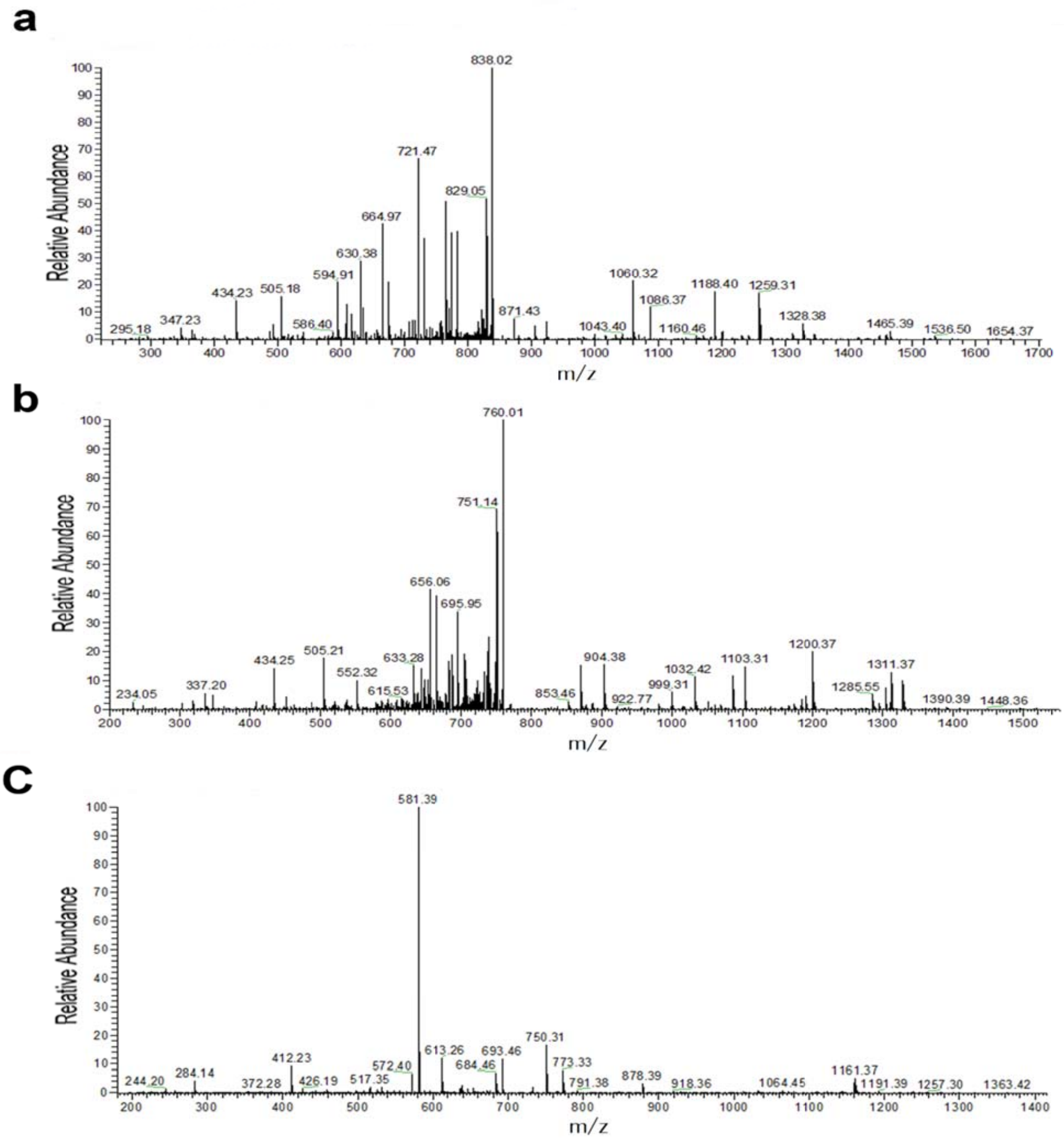

Figure S4. Mass spectrum profile of predigested Cy5 labeled Tat protein.

The purified Cy5 labeled Tat protein (The highest peak corresponding to the retention tim e of 7.86 min in Fig. S3) from HeLa cells was further analyzed by LC-MS spectroscopy. Identification of Tat proteins using LC-MS data relies on the peptide map from a Tat sequence target matching database. The following peptide sequences in Tat protein match with the spectrum measurement. (a) RAHQNSQTHQASLSK (b) AHQNSQTHQASLSK (c) LEPWKHPGSQPK, respectively.

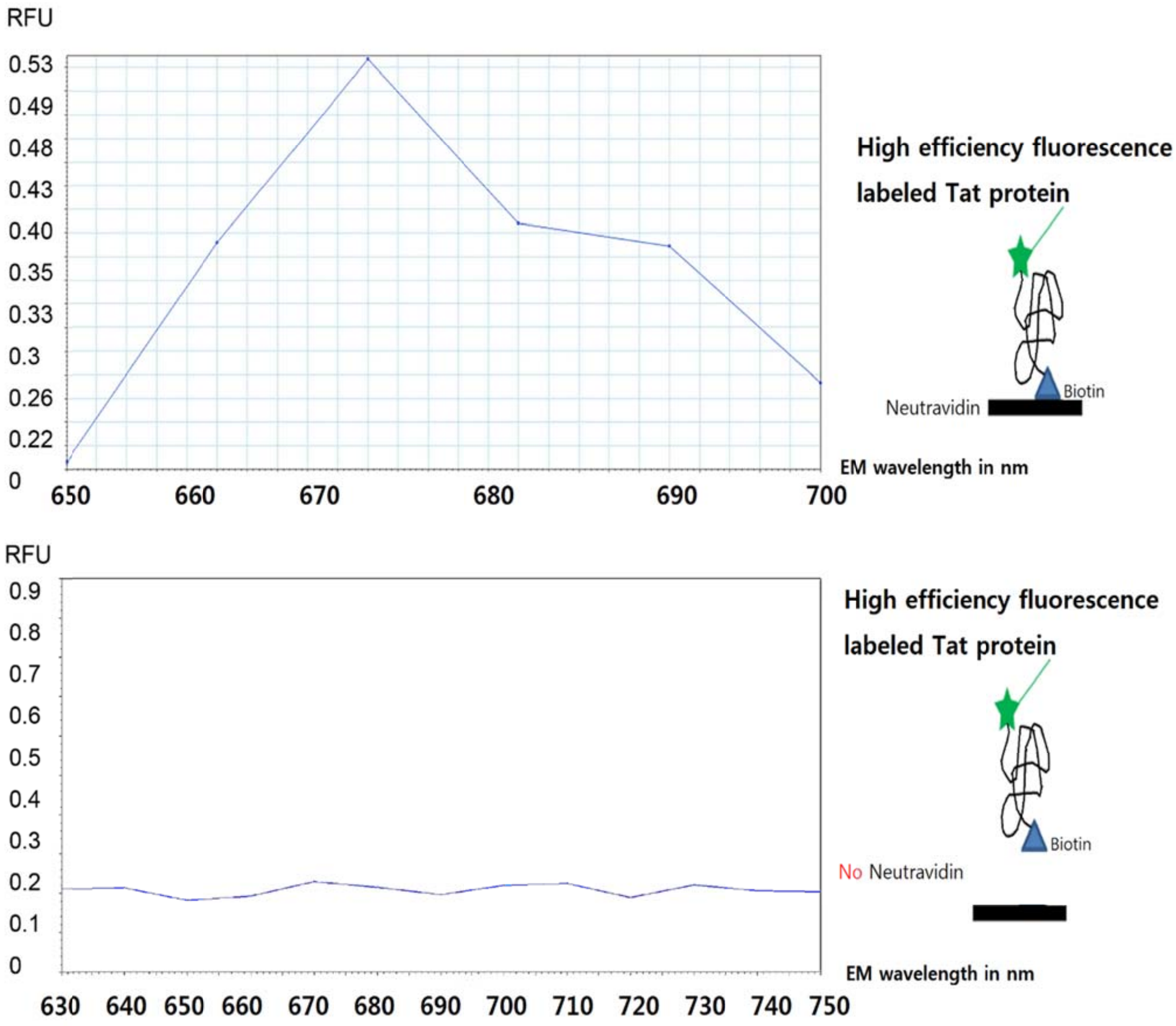

Figure S5. Evaluation of the Cy5 labeled HIV Tat protein for N-terminal fluorescent probe activity in HeLa cells by analytical fluorescence spectrum.

Detection of the N-terminally fluorescence-labeled Tat protein was performed using Neutravidin coated 96 well plates. Tat protein containing a C-terminal AVI-tag was recovered from HeLa extracts using Ni-affinity spin columns. Fluorescence spectrum wavelengths corresponding to the Cy5-labeled biotinylated Tat protein were analyzed using the fluorescence spectrum (Red laser; 620-670nm) detection. Positive control Fluorescence spectrum (green laser; 565-590nm) from ATTO 565-Biotin (Sigma), Negative control and 50 mM Phosphate Buffer pH7.4 are shown in Fig. S6.

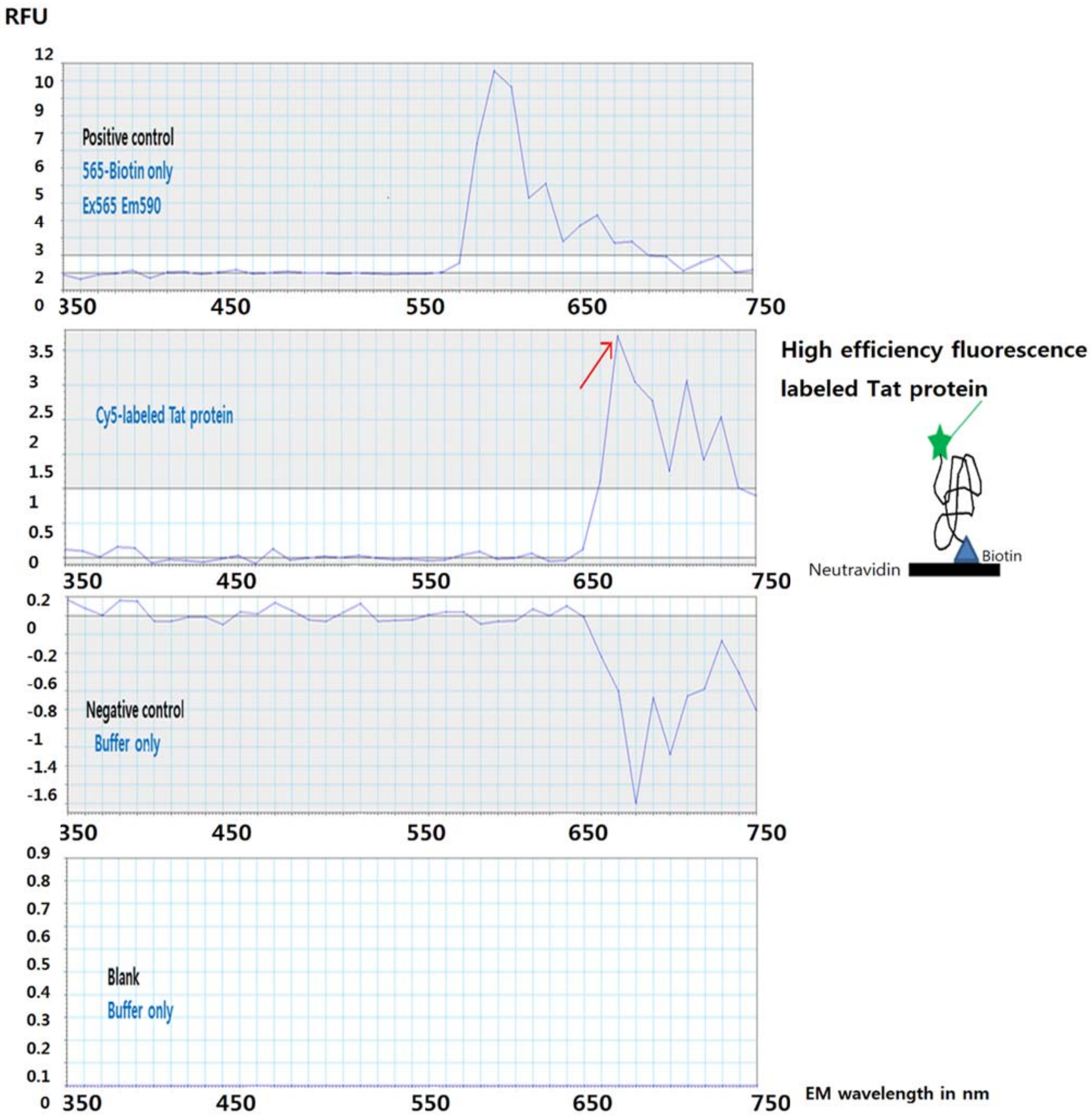

Figure S6. Evaluation of the Cy5 labeled HIV Tat protein while N-terminal fluorescent probe activity as control in HeLa cells by analytical fluorescence spectrum.

Detection of the N-terminally fluorescence-labeled Tat protein as control was performed using Neutravidin coated 96 well plates. Positive control Fluorescence spectrum (green laser; 565-590nm) from ATTO 565-Biotin (Sigma), Negative control and 50 mM Phosphate Buffer pH7.4.

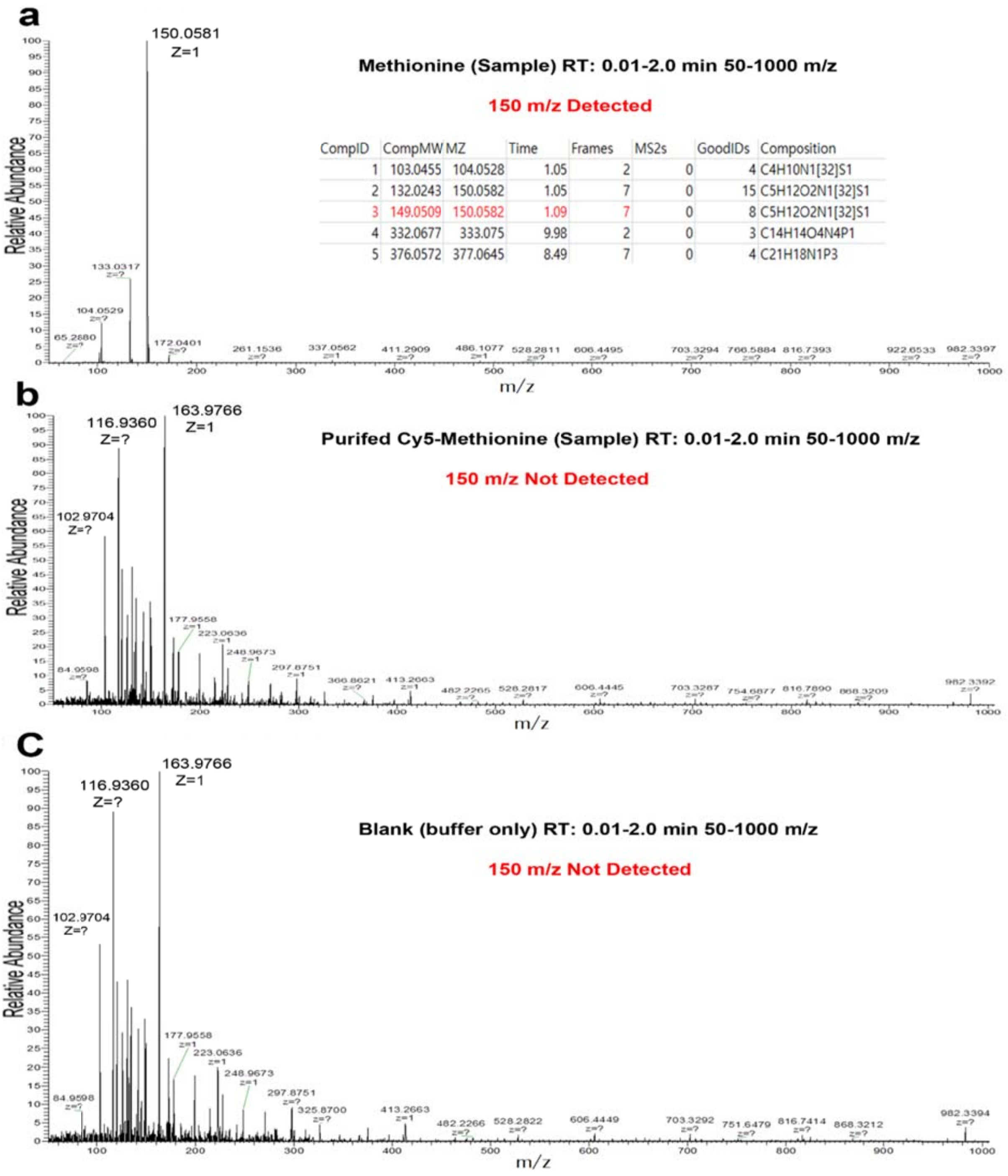

Figure S7. Comparison of LC-Mass spectrum profile of the purified Cy5 labeled methionine. (a)The liquid chromatography–mass spectrometry (LC-MS) of methionine alone (The highes t peak corresponding to the retention time (RT) of 0-2 min; 150 m/z detected) was further analyzed by LC-MS chemical matching database. The LC-MS spectrum of purified Cy5-Met (b) is almost identical to the corresponding fraction from buffer only control (c) (The highest peak corresponding to the RT of 0-2 min; 150 m/z not detected).
